# Emerging Prognostic Biomarkers in Testicular Germ Cell Tumors: Looking Beyond Established Practice

**DOI:** 10.3389/fonc.2018.00571

**Published:** 2018-11-28

**Authors:** Michal Chovanec, Costantine Albany, Michal Mego, Rodolfo Montironi, Alessia Cimadamore, Liang Cheng

**Affiliations:** ^1^2nd Department of Oncology, Faculty of Medicine, Comenius University and National Cancer Institute, Bratislava, Slovakia; ^2^Division of Hematology and Oncology, Indiana University Melvin and Bren Simon Cancer Center, Indianapolis, IN, United States; ^3^Section of Pathological Anatomy, Polytechnic University of the Marche Region, School of Medicine, United Hospitals, Ancona, Italy; ^4^Department of Pathology and Laboratory Medicine, Indiana University School of Medicine, Indianapolis, IN, United States; ^5^Department of Urology, Indiana University School of Medicine, Indianapolis, IN, United States

**Keywords:** testis, testicular germ cell tumors, molecular genetics, biomarkers, liquid biopsy

## Abstract

Testicular germ cell tumors are unique among solid cancers. Historically, this disease was deadly if progressed beyond the stage I. The implementation of cisplatin-based chemotherapy regimens has drastically changed the clinical outcome of metastatic testicular cancer. Several biomarkers were established to refine the prognosis by International Germ Cell Collaborative Group in 1997. Among these, the most significant were primary tumor site; metastatic sites, such as non-pulmonary visceral metastases; and the amplitude of serum tumor markers α-fetoprotein, β-chorionic gonadotropin, and lactate dehydrogenase. Since then, oncology has experienced discoveries of various molecular biomarkers to further refine the prognosis and treatment of malignancies. However, the ability to predict the prognosis and treatment response in germ cell tumors did not improve for many years. Clinical trials with novel targeting agents that were conducted in refractory germ cell tumor patients have proven to have negative outcomes. With the recent advances and developments, novel biomarkers emerge in the field of germ cell tumor oncology. This review article aims to summarize the current knowledge in the research of novel prognostic biomarkers in testicular germ cell tumors.

## Introduction

**Figure 1:**
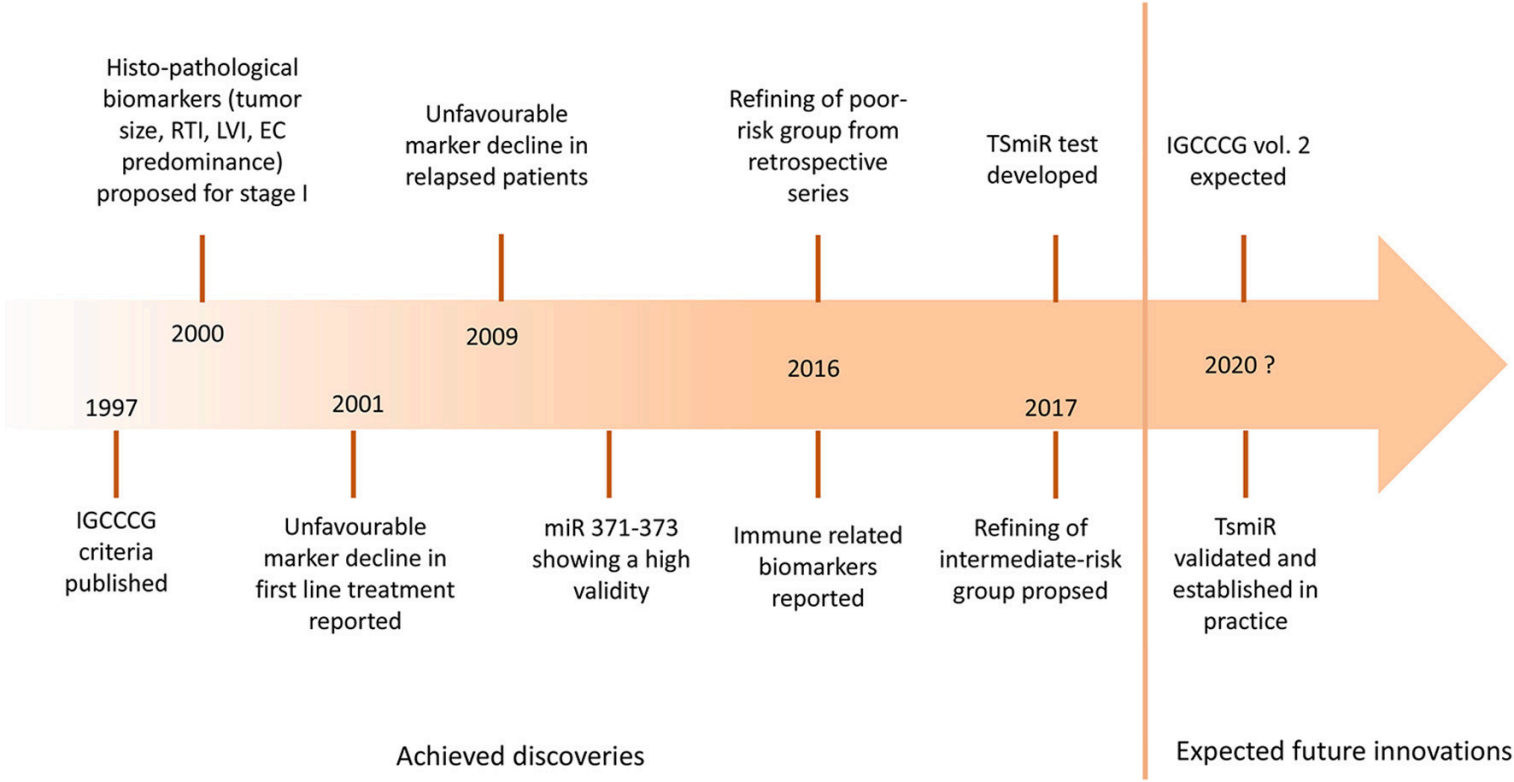
The landmarks of prognostic biomarkers in germ cell tumors. IGCCCG, International Germ Cell Cancer Consensus Group; RTI, rete testis invasion; LVI, lymphovascular invasion; EC, embryonal carcinoma; miR, microRNA; TSmiR, targeted serum microRNA test.

Testicular germ cell tumors (GCT) are unique in terms of molecular landscape, pathogenesis, clinical presentation, and response to chemotherapy (1). The exceptional position of GCT among the solid cancers can be perhaps attributed to their developmental origin in primordial germ cells. While the cure rate of patients with metastatic disease exceeds 80% (2), the ones failing the initial and salvage chemotherapy die of their disease in young age. About 40–80% of patients with relapsed GCT fail the salvage chemotherapy, resulting in the loss of 35 years of life on average (3–5). The utility of biomarkers to risk-stratify the treatment is well-established in GCT. Markers of the risk of relapse in the stage I disease, such as tumor size of >4 cm and rete testis invasion for seminoma, and lymphovascular invasion and predominance of embryonal carcinoma for non-seminoma, are currently used to risk-stratify the patients for surveillance or adjuvant treatment (6–9). International Germ Cell Cancer Collaborative Group (IGCCCG) presented the risk-stratification model for metastatic disease in 1997 using biomarkers such as primary tumor site, metastatic sites, the amplitude of serum α-fetoprotein (AFP), β-chorionic gonadotropin (HCG), and lactate dehydrogenase (LDH) (10). These criteria are based on patient series collected retrospectively between 1975 and 1990. Since then, the treatment strategy was optimized, and outcomes improved as reported from high volume centers (2, 11, 12). Further refining of IGCCCG criteria is expected soon in the updated version of the IGCCCG classification (Figure 1).

New reports on novel biomarkers are scarce since the introduction of the commonly used GCT biomarkers over three decades ago. The utility of novel molecular biomarkers in numerous solid cancers has significantly moved the advancement of oncology. Malignancies, such as lung cancer, melanoma, and kidney cancer, were previously considered untreatable, but now the array of molecular markers renders these diseases treatable with targeting agents ultimately prolonging lives of patients with incurable cancer (13, 14). Such advancement seemingly evades testicular GCT due to lack of known drugable targets. While the overall cure rate of GCT patients is excellent, ones refractory to standard chemotherapy lack the possibility to receive novel effective treatments and their prognosis is dismal. The biology of GCTs is unique, therefore translational research to uncover the biological implications is essential in the pursuit of treatment targets that may improve the prognosis of platinum refractory GCT patients. This article aims to summarize the current knowledge on the emerging biomarkers in GCT.

## Brief overview of molecular landscape in testicular germ cell tumors

Understanding why we lack a significant predictive biomarker in GCT requires a look into their molecular landscape and developmental origins. The origin of GCT particularly show how different their biology is compared to other solid cancers. The data from The Cancer Genome Atlas (TCGA) show a rather quiet mutational landscape in GCT compared to other solid tumors (15).

Several genomewide studies suggested driver mutations in only three genes (*KIT, KRAS*, and *NRAS*) in 4–31% of seminoma, and up to 14% of non-seminoma patients (16–19). Since these mutations were discovered in a minority of patients, a single universal mutational driver is not a feasible explanation in the development of GCT. Rather, a polygenic nature of testicular cancer was proposed, where the number of low frequency susceptibility genes (up to 50 risk loci reported until present) seems to produce an increased risk for the GCT (20). A recent paper by Shen et al. conducted a comprehensive molecular characterization of available tissue from 137 GCT patients. The authors confirmed findings of previously known mutated genes (*KIT, KRAS*, and *NRAS*) and provided yet additional evidence of low mutational burden with frequency of 0.5 mutations per megabase (15).

Despite the unimpressive mutational characteristics, GCT share a unique epigenetic landscape. GCT subtypes are an example of developmental processes from pluripotent embryonic stem cells toward certain degrees of differentiation to somatic tissues. The mapping of GCT methylome is perhaps the most comprehensively assessed to this date. The global DNA-methylation status clearly correlates with the state of differentiation in the histological GCT subtypes. Seminomas, which show the lowest degree of differentiation are typically unmethylated or severely hypomethylated tumors. Embryonal carcinomas show low to intermediate levels of global DNA methylation and well-differentiated yolk sac tumors, and teratomas show high levels of DNA methylation. Thus, the significant histological variability complies with the epigenetic heterogeneity. These findings also comply with the epigenetic landscape of healthy tissues where differentiated somatic tissues show hypermethylated pattern (21–23). Non-CpG methylation, acetylation, and methylation of histones are also mechanisms likely involved in the biology of GCT. They are, however, poorly understood in present time. microRNA (miR) signaling research on the other hand seems to provide promising results toward increasing the knowledge about molecular biology of GCT. While the miR signaling is generally complex and is a subject of innumerous interactions, the clusters of miR discussed later in this paper provide a significant biomarker potency to further refine the management of GCT.

The unique germline origin of GCT is underlined with the overexpression of markers of pluripotency such as NANOG, OCT3/4 or a tissue stem cell factor KIT and its' ligand (24–30). The expressions of these markers have been linked to epigenetic regulation with DNA methylation and histone acetylation (30–34).

## Emerging biomarkers in germ cell tumors

### Clinical biomarkers

IGCCCG vol. 2 will bring a long-awaited update for risk stratification of treatment of GCT based on clinical characteristics. The advent of clinical biomarkers is rather slow since the original publication of the IGCCCG criteria. Several other risk assessment criteria were proposed that considered a more detailed look into clinical characteristics in GCT patients. Adra et al. published results of their retrospective analysis of 273 patients with a poor risk disease treated at Indiana University (35). Primary mediastinal non-seminoma (PMNSGCT), brain metastases and increasing age were significant predictors of mortality (HR = 4.63, 3.30, and 1.06, respectively). Multiple criteria for a poor risk disease carried a significantly worse prognosis compared to a single criterion (35).

Necchi et al. proposed an improved model for intermediate risk patients in the two-institutional initiative using PMNSGCT, brain metastases, pulmonary metastases, and age at diagnosis as risk factors. According to the results, a number of intermediate risk patients would suffice from treatment with BEPx3, whereas the current standard remains BEPx4 (11). While the refining of prognosis based on clinical criteria may have reached its limits, authors from Memorial Sloan Kettering Cancer Center have proposed a novel prognostic marker based on a marker decline after the first course of chemotherapy (36). Patients who had unfavorable (slower) marker decline after the initiation if chemotherapy had reportedly worse outcomes compared to patients with favorable marker decline (72 vs. 95% for 2-year overall survival; *P* < 0.01) (36). These findings were subsequently replicated in independent studies (37, 38).

Furthermore, the prognostic significance of tumor marker decline was reported also in patients with relapse (39–41). Fizazi et al. conducted a randomized phase III study in poor risk GCT. Patients receiving first cycle of BEP had an assessment of serum markers prior to second cycle and ones with an unfavorable decline were randomized to receive either remaining three cycles of standard BEP or dose-intensified chemotherapy regimen. Based on this biomarker-based strategy, a significant advantage was reported for 5-year progression-free survival (PFS) (60 vs. 48%, *P* = 0.037), but not for 5-year overall survival (OS) (70 vs. 61%, *P* = 0.012) (42, 43). Interestingly, in cases of progression, patients from this study relapsed predominantly in brain (54% of all relapses) (44).

### Molecular biomarkers from immunohistochemistry studies

Immunohistochemistry studies have started to emerge in recent years to supplement the clinical biomarkers in predicting the prognosis of GCT. The higher expression of DNA repair enzyme poly (ADP-ribose) polymerase (PARP) was reported in GCT tissue compared to normal testicular tissue. However, no association with clinical characteristics nor the survival difference was reported in regard to levels of expression (45).

Kalavska et al. published two studies examining the prognostic value of carbonic anhydrase nine assessed from plasma and from tumor tissue (46, 47). Levels of this marker of hypoxia and aggressive tumor behavior correlated in plasma and in tumor tissue. High expression in tumor was associated with shorter PFS; however, the clinically more useful utility of plasmatic assessment failed to be prognostic in GCT (46, 47). The hepatocyte growth factor (HGF) and its receptor c-MET were investigated by immunohistochemistry in tumors and in cell-line culture. c-MET is a known proto-oncogene involved in tumor progression and metastasis. Authors of this study reported an abundant immunohistochemical expression in both seminomas and non-seminomas, particularly in epithelial structures of well-differentiated subtypes such as teratomas, yolk sac tumors, and choriocarcinomas. Upon the activation of c-MET in an NT2 cell line (embryonal carcinoma), the cells acquired a more robust ability to proliferate, migrate, and invade. This may create the rationale for further research; however, the clinical significance of this finding is currently unknown (48).

### Immune-related biomarkers

The discovery of novel immune-related biomarkers, programmed-death receptor and its ligand (PD-1 and PD-L1) in various cancers, led to a confirmation of active PD-1/PD-L1 signaling also in GCT by Fankhauser et al. (49). The authors conducted an immunohistochemistry study and showed a frequent PD-L1 expression in 479 GCT tissue samples. Both seminomas and non-seminomas exhibited a significant expression of PD-L1 (in 73% and 64% of patients, respectively) (49).

Another research team led by Mardiak et al. performed a similar study and scored the PD-L1 expression semi-quantitatively with multiplicative quick score. The scores were correlated with clinical outcome. Patients with low levels of PD-L1 expression had significantly better PFS (HR = 0.40; *P* = 0.008) and OS (HR = 0.43; *P* = 0.040) (50). Furthermore, the expression of PD-L1 on tumor infiltrating lymphocytes (TIL) proved to be highly predictive of outcome in a reverse manner. Patients with high PD-L1 expression on TIL had significantly better prognosis than patients with low PD-L1 TIL (51). The prognostic significance of TIL was earlier reported by Bols et al., who also performed the phenotyping of immune-cell infiltrates (52). However, the abundant expression of PD-L1 does not seem to be predictive of response to treatment with immune-checkpoint inhibitors.

A phase II study with anti-PD1 agent pembrolizumab provided data about insufficient anti-tumor activity in refractory patients with GCT (53). While several case reports documented possible responses to immune-check point inhibitor, these are likely due to concomitant treatment with chemotherapy (54–56). Another phase II study with anti-PD-L1 agent avelumab is currently ongoing, which will shed more light on single agent immunotherapy in refractory GCT (NCT03403777).

Currently, there is a level of uncertainty in predicting response according to PD-L1 expression levels. While several cancer types have proven to be sensitive to PD-1/PD-L1 blockade based on PD-L1 expression, PD-L1 negative tumors were described to respond to such treatment as well. On the other hand, the expression of PD-L1 in tumor and TIL in GCT signifies a vivid immunogenic microenvironment but fails to respond to immunotherapy according to our present knowledge. As such, PD-1/PD-L1 axis seems to be only a part of the involved immune machinery and we are lacking a deeper understanding. Shen et al. recently published findings of comprehensive molecular characterization of GCT and did not discover a significant neoantigen signal in GCT, thus the insufficient activity of immune check-point inhibitors in GCT may be partly explained by this fact and the presence of very low mutational load (15).

Two independent studies published simultaneously examined the role of a simple marker of proinflammatory macroenvironment, a systemic-immune infiltration index (SII) (57, 58). SII is calculated from total counts of neutrophils, lymphocytes, and platelets. Fankhauser et al. reported numerous markers associated with poor prognosis in GCT, including low hemoglobin and albumin, high leukocytes, neutrophils, CRP, neutrophil to lymphocyte ratio, and SII (58). At the same time, our study showed that high SII was associated with poor prognosis in two independent cohorts of GCT patients. We also evaluated a combined prognostic value of SII and PD-L1 expression on TIL. As a result, we identified patients who never experienced death nor a relapse if they exhibited low SII and high PD-L1 on TIL (57). Both studies reported the prognostic significance of SII being independent from the standard IGCCCG risk criteria. SII can be easily calculated from complete blood count performed prior to treatment and offers a simple tool to predict outcome in metastatic GCT. Poor prognosis in patients exhibiting high levels of SII also suggests that proinflammatory pathways likely unleashed by an aggressive tumor microenvironment may point to an unsuccessful struggle of the host immune system to overcome the tumor growth. Furthermore, signaling of proinflammatory cytokines, such as IFN-α2, IL-2Rα, or IL-16, was reported to be associated with poor risk clinical characteristics and inferior survival in GCT patients (59).

Nilius et al. recently reported that high expression of β-1,4-galactosyltransferase-I (B4GALT1) in peripheral T-lymphocytes is a marker of lower risk of relapse in GCT patients treated with salvage high-dose chemotherapy and peripheral stem cell transplant (HR = 0.66; 95% CI 0.45–0.97; *P* = 0.02) (60). T-cells were collected before the high-dose chemotherapy using the non-myeloablative chemotherapy and granulocyte growth factor (60). B4GALT1 is important for interaction and adhesion of immune cells and its role in disease control in stage I lung cancer has been established (61). This study supported their hypothesis of the importance of activated peripheral T cells in *in vitro* experiments by lectin stimulation of mononuclear cells with Concavalin A. As a result, B4GALT1 was upregulated, particularly in CD4^+^ cells and an antiinflammatory cytokine IL10 was significantly expressed. Interestingly, higher levels of IL10 from patient T cells were also associated with better outcome in GCT (60). Activated T cells, thus, seem to play an important role in cancer control.

### Liquid biopsies and epigenetic biomarkers

Sensitive and specific biomarkers indicating the presence of cancer that are assessed from peripheral blood represent an attractive and convenient approach in the diagnosis malignancies. Researchers recently published an array of articles showing that certain clusters of miR are highly informative of the presence of viable cancer in GCT patients (62–70). Serum examination for miR371-373 showed sensitivity of 98–100%, exceeding the sensitivity of the commonly used serum tumor markers AFP and HCG (71, 72). The targeted serum miRNA test (TSmiR) was developed and it seems to be very effective in predicting viable GCT after orchiectomy in clinical stage I patients or after chemotherapy in metastatic disease (72). The clinical utility of the TSmiR test is therefore very promising and clinicians may be expecting this novel biomarker to be implemented in the common practice in the near future (73). One possible utility of these highly sensitive miRNAs seems to be predicting the presence of a microscopic disease in clinical stage I GCTs. As such, these are likely to change the outlook over adjuvant treatment vs. surveillance. Another valuable input would be predicting the presence of viable cancer in post-chemotherapy residual masses, thus refining the need to perform often difficult surgeries in this setting. However, TSmiR does not identify teratoma components which still represent a diagnostic dilemma in the residual disease. Establishing the novel clinical practice stems from our ability to validate the utility of TSmiR in larger prospective cohorts of patients.

Majewski et al. assessed five patients with stage I seminoma and evaluated a possible role of liquid biopsy in identifying the presence of the tumor. The study showed promising results and identified candidate genes in whole blood prior to orchiectomy. This series is, however, too small to draw any conclusions and a larger study is suggested for validation (74).

A global DNA hypermethylation was proposed as one of the acting mechanisms in cisplatin resistance, the most frustrating challenge for oncologists treating GCT patients. *In vitro* epigenetic studies suggested that treatment with DNA demethylating agents may restore the sensitivity to cisplatin (75–77). In a study by Beyrouthy et al., a GCT cell-line treated with decitabine was resensitized to cisplatin (78). Based on these findings, Albany et al. performed a series of experiments in cell-line culture and patient-derived xenograft mouse model using a second-generation inhibitor of DNA-methyltransferase guadecitabine. Upon treatment of platinum resistant xenografts, a significant growth inhibition and even complete tumor regression was registered (79). An ongoing phase I trial using guadecitabine in combination with cisplatin in refractory GCT will shed more light on clinical significance of these promising findings (NCT02429466).

## Conclusion

The investigation for biomarkers in testicular cancer has been insufficient in the past, but with emerging data our knowledge it is built up with an increasing consistency. Such consistency is essential to generate experimental data and perform laboratory research which will ultimately lead to development of novel drugs with a promise to overcome the resistance to cisplatin.

## Author contributions

MC and LC contributed to conception and design. MC drafted the manuscript. CA, MM, RM, and AC contributed critical revision of the manuscript.

### Conflict of interest statement

The authors declare that the research was conducted in the absence of any commercial or financial relationships that could be construed as a potential conflict of interest.
